# Supplementary material for: A Preliminary Study: Evaluation of Oral Trazodone as a Strategy to Reduce Anesthetic Requirements in Bitches Undergoing Ovariectomy
Source: Animals (Basel). 2025 Mar 17;15(6):854. doi: 10.3390/ani15060854 (PMC11939430; doi:10.3390/ani15060854)
Supplement: Supplementary file 1 [file animals-15-00854-s001.zip › animals-3481656-supplementary.pdf]

Table S1: Intraoperative hemodinamyc and respiratory values.

	TRAZODONE GROUP							CONTROL GROUP						
**Variables**	**P**	**T0**	**T1**	**T2**	**T3**	**T4**	**D**	**P**	**T0**	**T1**	**T2**	**T3**	**T4**	**D**
**$F_{E'}$Sevo (%)**	--	2,13 ± 0,30	2,51 ± 0,18 [b]	2,48 ± 0,18 [b]	2,56 ± 0,15 [b]	2,51 ± 0,17 [b]	--	--	**2,46 ± 0,12 ***	2,57 ± 0,17	2,62 ± 0,35	2,51 ± 0,46	2,38 ± 0,35	--
**$EtCO_2$ (mmHg)**	--	46,12 ±4,85	42,87 ±2,03	41,12 ± 3,68	41,62 ± 2,13	44,50 ± 2,89	44,87 ± 4,45	--	44,25 ± 5,20	40,50 ± 2,39	**38,37 ± 4,27** [a]	40,00 ± 4,50	**40,25 ± 3,95 ***	**40,62 ± 3,25 ***
**HR (beats/ min)**	58,25 ± 13,16	71,87 ± 21,77	**87,12 ± 14,42** [a]	83,87 ± 14,02 [a]	75,62 ± 20,94	78,00 ± 17,19	82,12 ± 23,69 [a]	57,62 ± 17,16	80,25 ± 16,60 [a]	**83,00 ± 20,10** [a]	**94,62 ± 13,34** [a]	**84,00 ± 21,67** [a]	75,50 ± 17,98	**83,75 ± 16,49** [a]
**SAP (mmHg)**	111,28 ± 10,84	99,29 ± 17,58	**88,86 ± 9,08** [a]	106,57 ± 10,15	104,00 ± 18,81	93,57 ± 15,43	104,00 ± 17,19	114,62 ± 10,72	101,62 ± 10,46	**93,37 ± 18,43** [a]	113,25 ± 16,55[c]	108,37± 18,66	**93,25 ± 22,81** [a, d]	104,25 ± 15,48
**DAP (mmHg)**	80,86 ± 11,45	68,57 ± 20,47	**49,43 ± 11,83** [a]	70,00 ± 9,57 [c]	69,00 ± 12,29	**53,39 ± 14,66** [a]	75,43 ± 19,34 [c, f]	83,62 ± 13,36	73,37 ± 7,58	**55,75 ± 18,42** [a]	79,50 ± 14,37 [c]	74,00 ± 17,46	**59,50 ± 16,72** [a]	73,62 ± 16,98
**MAP (mmHg)**	87,71 ± 11,28	80,29 ± 17,64	**64,00 ± 13,32** [a]	76,57 ± 12,62	81,00 ± 17,94	**69,43 ± 14,01** [a]	83,71 ± 19,08 [c]	92,75 ± 12,84	82,00 ± 10,61	68,50 ± 15,11	88,50 ± 12,17	85,75 ± 16,50	**72,87 ± 21,14** [a]	83,75 ± 18,64
**Tempera-ture**	37,96 ± 0,75	37,31 ± 0,85	36,86 ± 1,16	36,79 ± 1,21	**36,67 ± 1,21** [a]	**36,47 ± 1,24** [a, b]	**36,37 ± 1,36** [a, b, c]	38,12 ± 0,53	37,34 ± 0,52	36,91 ± 0,81	36,69 ± 0,96	**36,46 ± 0,87** [a]	**36,34 ± 0,91** [a, b]	**36,26 ± 0,90** [a, b, c]

Table S1._Intraoperative hemodinamyc and respiratory values at different points in time along the experimental period in the trazodone (TG) and control group (CG). P: preinduction; T0: intubated; T1: tegument aperture; T2: right ovary resection; T3: left ovary resection; T4: tegument closening; D: recovery. Values given as a mean ± SD. $p < 0.05$: [a] vs. P, [b] vs. T0, [c] vs. T1, [d] vs. T2, [e] vs. T3, [f] vs. T4, * vs. TG at the same time.
